# Oral Erythroplakia and Speckled Leukoplakia: retrospective analysis of 13 cases

**DOI:** 10.1016/S1808-8694(15)30793-X

**Published:** 2015-10-19

**Authors:** Elaini Sickert Hosni, Fernanda Gonçalves Salum, Karen Cherubini, Liliane Soares Yurgel, Maria Antonia Zancanaro Figueiredo

**Affiliations:** 1 Master's degree in buccomaxillofacial surgery and traumatology (UFPEL). Doctoral student in clinical stomatology (PUCRS). Assistant professor, Universidade Federal de Pelotas; 2 Doctor in clinical stomatology. Adjunct professor, Pontifícia Universidade Católica do Rio Grande do Sul; 3 Doctor in clinical stomatology. Adjunct professor, Pontifícia Universidade Católica do Rio Grande do Sul; 4 Doctor in clinical stomatology. Adjunct professor, Pontifícia Universidade Católica do Rio Grande do Sul; 5 Doctor in clinical stomatology. Adjunct professor, Pontifícia Universidade Católica do Rio Grande do Sul. Pontifícia Universidade Católica Do Rio Grande Do Sul; Universidade Federal de Pelotas

**Keywords:** oral cancer, erythroplasia, risk factors, oral leukoplakia, oral mucosa

## Abstract

Erythroplakia and speckled leukoplakia are oral precancerous lesions that have a high potential for malignant transformation.

**Aim:**

A retrospective analysis was conducted to investigate the clinicopathologic features of 13 cases of oral erythroplakia and speckled leukoplakia in patients who were seen at a center specialized in stomatology and Histopathological diagnosis of oral diseases.

**Materials and Methods:**

All cases diagnosed with erythroplakia and speckled leukoplakia between 1978 and 2006 were retrieved from the service archives.

**Results:**

The lesions exhibited a predilection for males with a female-to-male ratio of 1:3.3. Mean age was 57 years old and soft palate was the site affected in 77% of the cases. Pain symptoms were reported by 61.5% of the patients and association with risk factors such as smoking and excessive alcohol intake was seen in 100% and in 46% of the cases, respectively. The lesions showed epithelial dysplasia, where more than 50% were diagnosed as in situ or invasive carcinoma.

**Conclusions:**

Despite low prevalence, oral homogeneous erythroplakia and speckled leukoplakia show Histopathological alterations vary from epithelial dysplasia to invasive carcinoma. These lesions must be included among those oral lesions with the highest potential for malignant tranformation.

## INTRODUCTION

The term oral erythroplakia is used to describe a red plaque or macular lesion in the mouth for which a specific clinical diagnosis cannot be established.^1^^,^^2^ Lesions are named erythroleukoplakia, leukoerythroplakia or speckled leukoplakia when red and white areas are associated or white patches are present over the red plaque.^2^^,^^3^ The World Health Organization (WHO)4 currently employs the term speckled leukoplakia to describe mouth lesions that present erythroplasic and leukoplasic components; this will be the term used in this study.

Oral erythroplasia is rare, but its malignant transformation rate is the highest among all of the precancerous lesions in the mouth;^5^^,^^6^ dysplasia, in situ carcinoma or invasive carcinoma may be found in over 90% of cases.^7^, ^8^, ^9^ Although the potential for malignant transformation is higher in erythroplakia, speckled leukoplakia should not be neglected, since the red patches in this lesion have a similar histology to homogeneous erythroplakia.^10^, ^11^, ^12^, ^13^

The purpose of this study was to conduct a retrospective analysis of the clinical and pathological features of 13 cases of oral erythroplakia and speckled leukoplakia; the patients were seen at a clinic that specializes in stomatology and the histopathological diagnosis of mouth diseases.

## MATERIAL AND METHOD

This was a cross-sectional historical cohort study reviewing 17 831 files of patients with stomatological lesions biopsied from 1978 to 2006. Patients with a clinical diagnosis of erythroplakia or speckled leukoplakia were studied further to gather data on age, sex, tobacco and alcohol consumption, and the pain, site, size, duration and histopathological features of lesions. Descriptive statistics were used for data analysis.

## RESULTS

**Figure 1:**
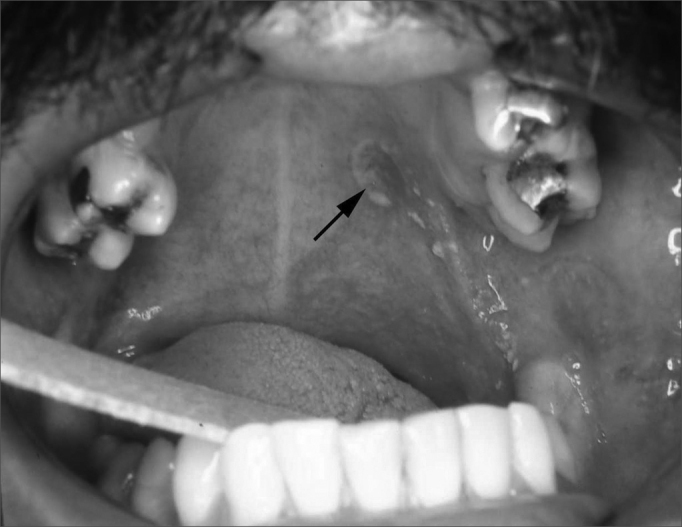
Homogeneous erythroplakia located in the soft palate. The ulcer (arrow) is the incision biopsy site.

**Figure 2:**
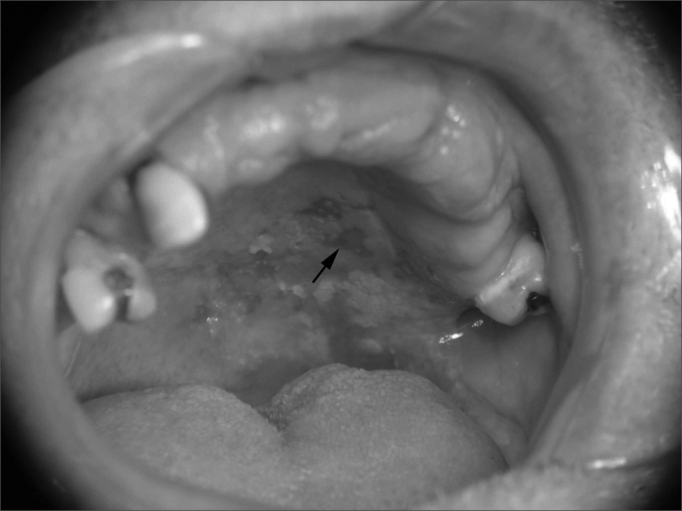
Speckled leukoplakia located in the soft and hard palate. Histopathology revealed acanthosis and hyperkeratosis in the leukoplasic area, and invasive carcinoma in the erythroplasic area (arrow).

Among 17 831 cases in the biopsy registers, 13 fulfilled the requirements for being included in this study. Two of these cases were diagnosed with homogeneous erythroplakia (Fig. 1) and eleven were diagnosed with speckled leukoplakia (Fig. 2). These lesions comprised 0.072% of the oral lesions in all patients seen at the stomatology clinic. Table 1 shows the data.

**Table 1:** Clinical and pathological features of 13 cases of homogeneous erythroplakia and speckled leukoplakia in patients seen from 1978 to 2006. Porto Alegre, 2007.

Sex	Age	Clinical Diagnosis	Histopathological Diagnosis.	Site	Symptoms	Smoking	Alcohol
M	55	Homogeneous erythroplakia	epithelial dysplasia	soft and hard palate	Pain	yes	yes
F	71	Homogeneous erythroplakia	epithelial dysplasia	soft palate	dysphagia	yes	no
M	33	Speckled leukoplakia	squamous cell carcinoma	ventral area of tongue	pain	yes	yes
M	39	Speckled leukoplakia	squamous cell carcinoma	soft palate and pillar	asymptomatic	yes	yes
F	42	Speckled leukoplakia	squamous cell carcinoma	floor	asymptomatic	yes	yes
M	48	Speckled leukoplakia	epithelial dysplasia	soft palate and pillar	dysphagia	yes	no
M	54	Speckled leukoplakia	squamous cell carcinoma	soft palate	dysphagia	yes	yes
M	62	Speckled leukoplakia	squamous cell carcinoma	soft and hard palate	asymptomatic	yes	no
M	62	Speckled leukoplakia	in situ carcinoma	soft palate and pillar	dysphagia	yes	no
M	66	Speckled leukoplakia	squamous cell carcinoma	soft palate	dysphagia	yes	yes
M	69	Speckled leukoplakia	in situ carcinoma	soft palate and pillar	asymptomatic	yes	no
F	70	Speckled leukoplakia	epithelial dysplasia	floor	asymptomatic	yes	no
M	71	Speckled leukoplakia	epithelial dysplasia	soft palate and pillar	pain	yes	yes

Males predominated in a 1:3.3 proportion. The age of the 13 patients ranged from 33 to 71 years; the mean age was 57 years (SD – 13.08). The soft palate was involved in 77% of erythroplakia and speckled leukoplakia cases (10 patients); in 70% of these (7 cases) the lesions also involved the hard palate or the tonsillary pillar. The diameter of lesions ranged from 1.5 cm to 4 cm, a mean 2.58 cm (SD – 0.87). Pain, with or with no dysphagia, was reported by 61.5% of patients; the remaining patients were asymptomatic.

Smokers comprised 69.2% of the 13 patients. All of the remaining patients had a history of smoking, that is, they had smoked during at least five years but had ceased smoking during the period in which lesions were developing. Excessive alcohol consumption was reported by 46% of patients. Candida infection overlapping the lesions was suspected in 61.5% of patients; in these cases, oral nistatin mouthwashes were prescribed before incision biopsies.

**Figure 3:**
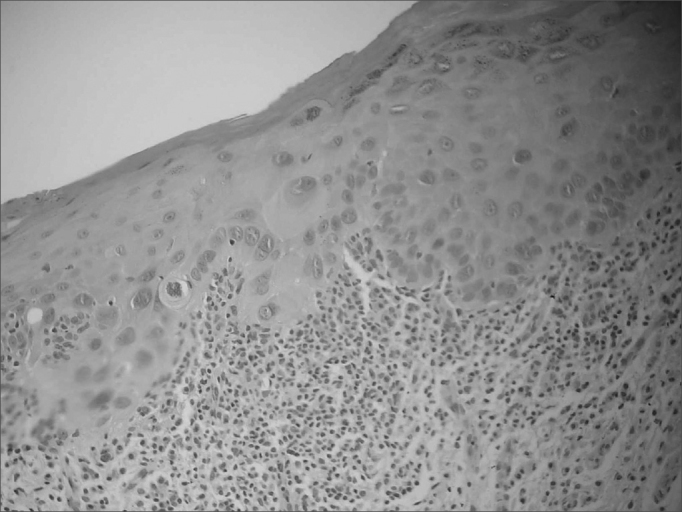
Histopathology of a speckled leukoplakia case showing moderate epithelial dysplasia (H&E, 200X).

Biopsy material was taken from both white and red patches when these were present concomitantly on lesions. Some degree of dysplasia was seen in all of the red patches. The histopathological diagnosis of speckled leukoplakia cases was epithelial dysplasia in 27% (n=3) of cases (Fig. 3), in situ carcinoma in 18% (n=2) of cases, and invasive carcinoma in 55% (n=6) of cases. The diagnosis was epithelial dysplasia in the two cases of homogeneous erythroplakia. The histopathological diagnosis of an erythroplasic patch in a cases of speckled leukoplakia was grade II squamous cell carcinoma; in this same case, the leukoplasic patch was diagnosed as having acanthosis and hyperkeratosis.

Surgical resection of lesions was done in four of eight patients with a diagnosis of in situ or invasive carcinoma; radiotherapy was carried out in the other four cases. Surgical resection was done in two patients with a diagnosis of epithelial dysplasia. Two other patients continued to be monitored clinically; their lesions have regressed partially after smoking was ceased. One patient did not return for treatment.

## DISCUSSION

Erythroplakia and speckled leukoplakia are uncommon lesions of the mouth. From 1978 to 2006, 13 biopsied cases of 17 831 patients with stomatological lesions had homogeneous erythroplakia or speckled leukoplakia. The prevalence of these lesions in this study was below the 0.4% reported by Mallo-Pérez et al.,^14^ who studied elderly institutionalized patients. We reviewed patients belonging to all age groups. Lapthanasupkul et al.^15^ investigated precursor oral lesions in a group of Thai patients and found a prevalence rate of 0.17%; this is possibly due to regional differences, especially smoking.

The results of an analysis of the clinical features of these lesions are similar to other published findings in terms of the sex and symptoms of patients and preference for the soft palate; in our study, 10 of 13 cases presented lesions on this site.^5^^,^^14^^,^^15^ Eight of the 13 patients were aged between the 6th and 8th decades, and two patients were in the 5th decade of life; this is similar to other published results for oral erythroplakia and speckled leukoplakia. Lesions developed in younger patients in two cases (aged 33 and 39 years); both patients were smokers and consumed alcohol. Histopathology in these cases revealed squamous cell carcinoma. Although the main risk factors were present, lesions in these younger subjects may also have been associated with inherent factors, such as genetic mutation.

Risk factors for oral carcinoma, such as alcohol use and smoking, diets lacking antioxidants (such as vitamins C, E, and beta-carotenes), occupational exposure to carcinogens, viral infections, and genetic and hereditary factors, may affect how these precancerous lesions become established and develop.^16^, ^17^, ^18^, ^19^ In our study we were unable, however, to analyze risk factors other than smoking and alcohol use, since we conducted a retrospective investigation of files, many of which did not contain such information. Smoking was the most prevalent of the factors above that we were able to study; there was a history of smoking in all cases. Consumption of alcohol was associated with 46% of cases. No patient had a history of occupation exposure to carcinogens.

Biopsies were taken of both the erythroplasic and leukoplasic areas in cases of speckled leukoplakia. Some degree of epithelial dysplasia was present in all of the red patches; the histopathological diagnoses ranged from dysplasia to invasive squamous cell carcinoma. In one case, different histopathological findings were found in the same lesion: there was no dysplasia in the leukoplasic area, and malignancy in the erythroplasic area. This finding underlines the need for taking biopsies of various areas in cases of speckled leukoplakia, including the erythroplasic component. Pindborg et al.^9^ investigated mouth carcinomas and found that 64% of cases arose from areas of speckled leukoplakia. Banoczy & Csiba10 and Banoczy12 reported that 26% of carcinomas developed over sites of speckled leukoplakia, while only 2% developed from other types of leukoplakia.

Depending on the histopathological diagnosis, a possible approach in patients with erythroplakia or speckled leukoplakia would be to monitor some patients clinically and to carry out periodic incision biopsies.^20^^,^^21^ This strategy was applied to two of our patients, since they presented extensive speckled leukoplakia and the histopathological diagnosis was mild epithelial dysplasia. These patients complied with therapy, which included periodic return visits, sequential biopsies, and cessation of smoking and alcohol consumption. Surgical excision is the treatment of choice made by most healthcare professionals; it was done in six of the 13 cases. Other treatments have been proposed, such as: topical retinoic acid with systemic beta-carotens;^22^ photodynamic therapy with methyl aminolevulinate;^23^ and cryosurgery or vaporization with carbon dioxide laser radiation.^24^ These approaches also include cessation of risk factors. In cases that have progressed to carcinoma, surgery (followed or not by radiotherapy), radiotherapy, and chemotherapy have been the usual approaches.^21^

Reichart & Philipsen^6^ have stated that only homogeneous red erythroplakia has been clearly defined, while the terminology for mixed lesions is confusing; doubts remain about how to quantify the red and white patches. These authors have also suggested that the natural history of homogeneous erythroplakia is unknown; it is not clear whether lesions develop de novo or from preexisting leukoplakia.^6^ As shown above, various designations have been used to describe the presence of both white and red patches. We chose the term “speckled leukoplakia” in this study to standardize the nomenclature; this term currently used by the WHO.^4^

## CONCLUSION

The clinical and pathological features of the lesions analyzed in this study support the data in other published studies. Although their prevalence is low, homogeneous erythroplakia and speckled leukoplakia present histopathological features ranging from epithelial dysplasia to invasive carcinoma. This justifies placing these lesions among the oral lesions with the highest malignant potential. Additionally, regardless of histopathology and therapy, periodic monitoring of these patients and cessation of risk factors are essential measures in such cases.
